# Metal/Perovskite Plasmonic–Photonic Heterostructures for Active and Passive Detection Devices

**DOI:** 10.3390/mi16040424

**Published:** 2025-04-01

**Authors:** Dominik Kowal, Yuntian Chen, Muhammad Danang Birowosuto

**Affiliations:** 1 Łukasiewicz Research Network—PORT Polish Center for Technology Development, Stabłowicka 147, 54-066 Wrocław, Poland; muhammad.birowosuto@port.lukasiewicz.gov.pl; 2 School of Optical and Electronic Information, Huazhong University of Science and Technology, Wuhan 430074, China; yuntian@hust.edu.cn

**Keywords:** photonics, plasmonics, perovskites, nanostructures, photodetector

## Abstract

Recent advancements in metal/perovskite photodetectors have leveraged plasmonic effects to enhance the efficiency of photogenerated carrier separation. In this work, we present an innovative approach to designing heterostructure photodetectors that involved integrating a perovskite film with a plasmonic metasurface. Using finite-difference time-domain (FDTD) simulations, we investigated the formation of hybrid photonic–plasmonic modes and examined their quality factors in relation to loss mechanisms. Our results demonstrate that these hybrid modes facilitated strong light confinement within the perovskite layer, with significant intensity enhancement at the metal–perovskite interface—an ideal condition for efficient charge carrier generation. We also propose the use of low-bandgap perovskites for direct infrared passive detection and explore the potential of highly Stokes-shifted perovskites for active detection applications, including ultraviolet and X-ray radiation.

## 1. Introduction

Metal–semiconductor heterostructures utilizing a Schottky barrier $\Phi_{B}$ for photogenerated charge carrier separation offer many advantages for light detection applications [1,2,3,4], including high-speed operation, a broadband response, tunability, design simplicity and the possibility of on-chip integration. However, they also suffer from the strong reflection of incoming light at the metal interface and a narrow depletion region, limiting the efficiency of light absorption and carrier generation. In recent years, metallic nanostructures that support various kinds of plasmonic modes were explored to address these challenges. Localized surface plasmon resonances (LSPRs) or traveling surface plasmon polaritons (SPPs) excited at the metal/semiconductor interface can produce very tight light confinement that can be utilized to efficiently generate charge carriers in both the metal and semiconductor through internal photoemission (IPE) and electron–hole creation, respectively. Alavirad et al. [5] reported a 13 mA/W responsivity at a 1.55 μm telecom wavelength of plasmon-enhanced photodetector utilizing Au nanograting on p-type Si. Frydendahl et al. [6] demonstrated 4.5 mA/W responsivity at 1.3 μm and a 2 mA/W responsivity at 1.55 μm using thin-film Al on p-type Si, which showed a two orders of magnitude responsivity enhancement due to plasmonic effects. The reported plasmon-enhanced junctions typically use n- or p-type Si with a plasmonic metal contact, such as Au, Ag or Al [1,5,6,7]. Alternatively, Wang et al. [8] demonstrated a device based on Ge/Au nanograting, which led to a higher responsivity of 0.38 A/W at 1.55 μm and a detection band that extended to 1.9 μm. Several works explored the potential of two-dimensional materials, including graphene or transition metal chalcogenides, for an improved junction performance [2,3,9].

As a potential alternative to Si and Ge, some researchers attempted to use halide perovskites. In many aspects, perovskite materials show optoelectronic properties superior to those of Si, especially where a tunable bandgap, high absorption coefficients, an active operation scheme and mechanical flexibility are desired. In active devices, the perovskites offer advantages due to their very good emission properties in the visible or infrared range and high refractive index values. Also, convenient processing techniques can be used for making perovskite-based nanostructures or nanocomposites that can be tailored for specific emission characteristics or emission improvement through effects such as Purcell enhancement [10,11] and nonlinearity [12]. Plasmonic enhancements have been widely used in perovskite optoelectronic devices [13] and there are several works that have reported the use of metal/perovskite junctions for photodetection based on materials such as MAPbI_3_ [14,15,16] or ${\mathrm{FA}}_{1-x}{\mathrm{Cs}}_{x}{\mathrm{PbI}}_{3}$ [17]. Zeng et al. reported 313 mA/W at a 800 nm wavelength in a MAPbI_3_/PdSe_2_ heterostructure and a broad detection band from 200 nm to 1.55 μm [17].

Schottky-type photodetectors with plasmonic enhancement were designed either in free-space operation mode [5,18] to respond to an external light signal or by utilizing integrated optical architectures based on the waveguiding mechanism [19,20]. In the case of free-space operation, the coupling of the incident light to plasmonic modes usually requires the presence of one- or two-dimensional grating with subwavelength dimensions to increase the in-plane wavevector. In the case of waveguided light, the coupling operation is more straightforward and can be achieved with simple planar geometry. In this work, we introduce a novel design approach to metal–semiconductor photodetectors based on the guided propagation of hybrid plasmonic–photonic modes in a perovskite thin film coupled with a plasmonic metasurface. The guided modes can be excited either in a passive scheme through the scattering of the external light beam on the plasmonic nanoarray or through active operation by leveraging perovskite photoluminescence. The metasurface geometry was designed to produce strong field localization in the near-infrared wavelengths around 1000 nm (*f* = 300 THz); however, the spectrum of detection can be tuned to shorter wavelengths by utilizing the active waveguide operation of the perovskite film. The advantage of using hybrid modes is that they can produce strong field enhancement at the metal/perovskite interface, while at the same time, the light absorbed at a greater distance from the interface might also contribute to the detected signal through energy sharing between the photonic and plasmonic components. These characteristics are useful for the design of thicker and possibly multi-band plasmonic Schottky photodetectors through the combination of passive and active layers that can work simultaneously. Utilizing the coupled optical response of the multilayered system is a new approach in photodetection that does not require switching between forward and reverse modes when combining the active and passive responses of the device [21]. The presented study utilized finite-difference time-domain (FDTD) simulations to explore the characteristics of the proposed heterostructures, including band dispersion diagrams, mode profiles and the influence of material absorption. Finite structures with dimensions up to 6.5×6.5μm were also considered to examine the intensity enhancement in what can represent a single detector pixel.

## 2. Design of the Hybrid Metasurface

**Figure 1:**
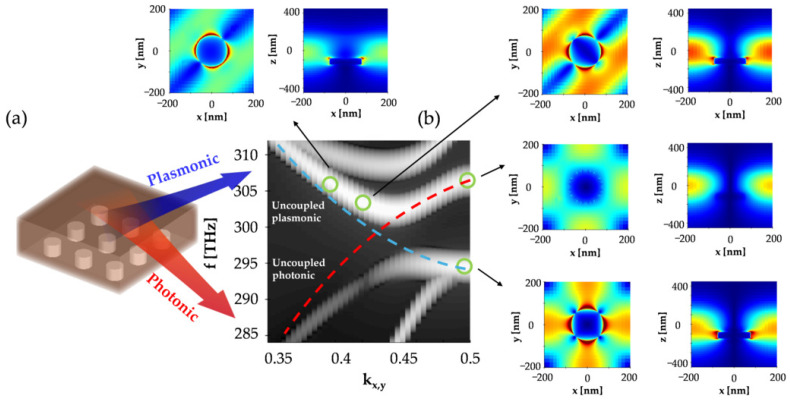
The formation of hybrid plasmonic–photonic modes in a thin film of perovskite coupled with an array of plasmonic nanopillars. (**a**) Schematic view of the hybrid metasurface, and (**b**) its local photonic band structure in the proximity of a high symmetry point M ($k_{x,y}$ = 0.5), with the insets showing the mode distributions along the higher and lower frequency bands. The dashed lines demonstrate the shape of the uncoupled photonic (red) and plasmonic (blue) bands.

The initial study took into consideration an array of silver nanopillars inside a thin film overlay with a thickness H= 300 nm and refractive index n= 2 on a bulk glass substrate. The pillars were arranged in a square lattice with a spatial period *a* and they were characterized by a radius *r* and height *d*. The presence of pillars resulted in periodic modulation of the overlay thickness, which led to the formation of guided Bloch modes with a characteristic frequency ω and in-plane wavevector $k_{\left|\right|}=\left(k_{x},k_{y}\right)$. The modulation strength depended on the ratio of the pillar height and overlay thickness d/H, which we set to 0.3. In addition to the Bloch modes traveling in the overlay (we refer to these modes as photonic), the nanopillars support surface lattice resonances (SLRs) that result from far-field coupling of the individual LSPRs [22] that are closely confined at the pillar surface. Nanopillars are a common choice for a plasmonic nanostructure geometry when generating SLRs [23]. Although other geometries, such as nanocubes or nanowires, can produce stronger field localizations and Q-factors [24], nanopillars are easier to fabricate [25] and more resistant to fabrication imperfections. Also, they usually generate a moderate resonance linewidth that is convenient for photodetection applications. In the case of significant overlap between the photonic and plasmonic modes, provided a symmetry and polarization match, the modes will experience interference, which manifests itself by avoiding the crossing of the two bands in the band dispersion diagram ω(k) [26]. Assuming infinite dimensions of the structure, we modeled the band dispersion and mode profiles with an FDTD simulation, and the results are presented in Figure 1. At this stage, for simplicity and clear observation of the mode behavior, the overlay was considered lossless and the pillars were isolated from each other (Figure 1a). The geometric parameters a= 400 nm, r= 90 nm, d= 90 nm and H= 300 nm were chosen to observe the crossing near 300 THz, with a large value of $k_{\left|\right|}$ close to the corner of the Brillouin zone *M*. As shown in Figure 1b, away from the crossing, the modes had predominant photonic or plasmonic characteristics; however, near the intersection, they became coupled, which gave rise to hybrid modes, with a significant contribution of both the plasmonic and photonic parts. Due to coupling, the energy stored in such a mode was not bound to the photonic or plasmonic part, but could be transferred between them over time, as was previously shown on the basis of coupled mode theory for plasmonic resonators integrated in waveguide structures [27,28]. This fact is particularly interesting in the context of designing plasmonic Schottky junctions for the purpose of light detection, where the dominant part of charge carriers is generated through absorption in a thin depletion region. SLR features are responsible for enhancing the field intensity in this region, which can be sustained for a longer time because of the interaction with the coupled photonic mode.

For the practical implementation of the above concept in designing the photodetector junction, the pillars should be interconnected with a thin silver patch that acts as an electrical contact, and the overlay must be made of a semiconducting material. The n= 2 refractive index may correspond to various kinds of perovskite materials, which can have different optoelectronic properties, including absorption, emission and carrier mobility. Through doping or synthesis modifications, perovskites can be made n- or p-type, which affects the charge carrier type and efficiency of the charge carrier mechanisms at the junction. Depending on the photon energy ℏω, electronic bandgap $E_{g}$ of the perovskite, and $\Phi_{B}$ at the junction for electrons and holes, there can be varying contributions of carriers injected from the perovskite to the Ag and vice versa. The former is associated with the band-to-band excitation of carriers and requires ℏω > $E_{g}$, while the latter results from IPE, which allows for sub-bandgap photon absorption (ℏω > $\Phi_{B}$).

**Figure 2:**
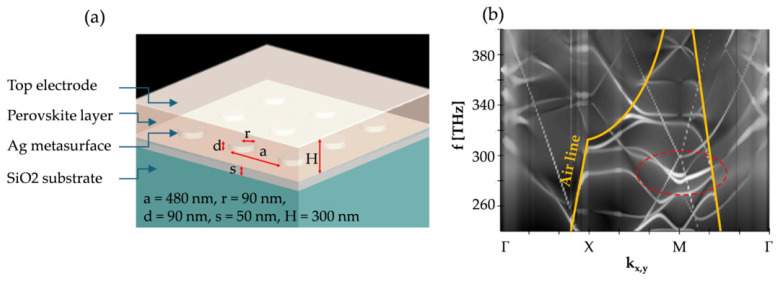
(**a**) Design of the plasmonic–photonic heterostructure proposed as the photodetector and (**b**) the corresponding band structure of the electromagnetic modes supported in it. The orange line represents the air line, which separated the guided and radiative mode regions. The red dashed line marks the mode of interest, which dominated other modes in the 290–300 THz frequency range.

This study focused mostly on the optical behavior of the structures, and so the overlay material was simply characterized by *n* and κ (see Section 4 for a more comprehensive discussion of the specific materials and applications). The top electrode of the junction can be made of a thin metal; transparent conductive oxides; polymers; or composite materials, such as transparent electrodes based on silver nanowires. Also, it can uniformly cover the overlay upper surface or can be positioned on the sides. In the presented FDTD simulations, the top electrode was not included, as it did not play a significant role in the demonstrated effects. Figure 2 shows the schematic design and the mode dispersion diagram of the proposed plasmonic–photonic heterostructure. In the guided region (below the light line) at the frequencies 280–300 THz, one can identify two modes that exhibited a hybrid plasmonic–photonic character and they dominated the band structure in the specified frequency range. The marked zone on the band diagram is analogical to the crossing sketched in Figure 1; however, the presence of silver patch made the band configuration more complex due to the formation of additional SPP modes. The demonstrated characteristics of the heterostructure imply that 280–300 THz radiation with a high enough $k_{\left|\right|}$ will most favorably couple with the hybrid modes and this can be exploited to manage the optoelectronic properties of the junction.

## 3. Field Confinement and Influence of Loss

**Figure 3:**
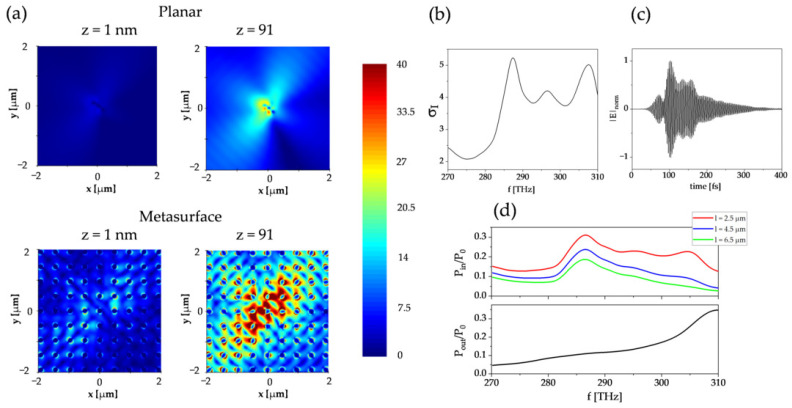
The demonstration of enhancement in the finite structure. (**a**) Comparison of the electric field magnitude |E| simulated in the planar (top) and structured (bottom) junction configurations at the vertical coordinate *z* = 1 nm, which corresponded to the metal patch, and *z* = 91 nm, which corresponded to the top of the pillars. (**b**) Intensity enhancement at the metal/perovskite interface in the metasurface junction (compared with the planar junction) integrated over the 270–310 THz frequency band and spatial coordinates. (**c**) Time dependence of the electric field at the metal/perovskite interface in the metasurface junction. (**d**) The ratio of optical power propagating in the overlay to the total power emitted at three different distances from the emitting dipole (upper) and the ratio of the out-of-plane radiating power to the total power emitted (lower) as a function of the frequency.

The above findings were then verified by simulating the light behavior in the structures with finite *x* and *y* dimensions. Figure 3a compares the magnitude of the electric field |E| that resulted from the local excitation of the junction with a designed metasurface architecture and reference planar architecture. Within the chosen spectral range of 270–310 THz, we found an average intensity enhancement $\sigma_{I}^{av}$ near the metal/perovskite interface as a factor of 3.6, where the enhancement spectrum showed a maxima up to $\sigma_{I}$ = 5 at frequencies of 286 THz and 307 THz (Figure 3b). In addition to the spatial field confinement, one observes the temporal confinement manifested by the prolonged decay of the electric field (Figure 3c). The excitation source was switched off at $t_{1}$ = 120 fs; however, the electric field recorded in the simulation was sustained until $t_{2}$ = 400 fs. The temporal confinement resulted from the excitation of hybrid modes with a low group velocity number (as observed in the simulated band structure, Figure 2b). The in-plane propagation and out-of-plane radiation from the structure were also examined in the simulation. The ratio of the optical power guided inside the overlay to the total power emitted $\frac{P_{in}}{P_{0}}$ had maxima that corresponded to the $\sigma_{I}$ peaks. As the wave propagated, the power was decreased due to the absorption and radiation losses (Figure 3d). In the current analysis, we kept the extinction coefficient of the overlay as κ = 0, so all the absorption was caused by the presence of silver. This was the reason for the loss of power in the low-frequency band of $\sigma_{I}$. In contrast, the high-frequency band was both absorbed and partially coupled with the free-space radiation, as observed in the optical power ratio radiated outside of the structure $\frac{P_{out}}{P_{0}}$. This implied that the high-frequency peak corresponded to the formation of leaky modes, and such modes could be observed in the computed band diagram at smaller wavevector numbers near the central point Γ.

**Figure 4:**
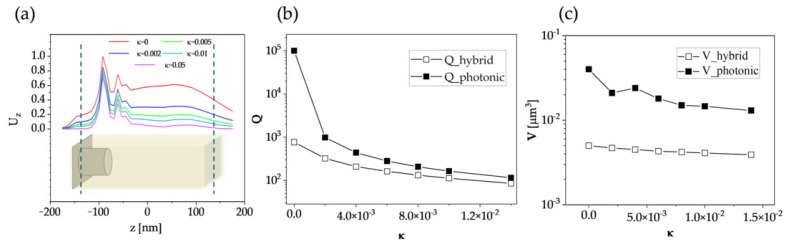
The effect of material absorption κ on the characteristics of the hybrid modes. (**a**) Simulated electromagnetic energy density $U_{z}$ along the vertical coordinate *z* in the heterostructure with varying absorption. The drops in the simulated (**b**) quality factor *Q* and (**c**) mode volume *V* with material absorption simulated for the hybrid mode and for the reference photonic mode.

To better understand the potential of the designed heterostructure in real applications, we also introduced the nonzero extinction coefficient κ of the overlay material and examined its significance to the formation of hybrid modes. Figure 4a shows the computed energy density $U_{z}$ carried by a mode as a function of the vertical coordinate *z* and integrated over the *x* and *y* coordinates and frequency *f*:

(1) $$U_{z}=\frac{1}{2}\times \;\int \int \int \;\left(\varepsilon |E{|}^{2}+\mu |H{|}^{2}\right)\;dx\;dy\;df,$$

where |E| and |H| are the electric and magnetic field magnitudes and ε and μ are the absolute permittivity and permeability of the overlay material. As expected, a big part of the energy was indeed confined near the upper surface of the nanopillars due to SLR effects. The contributions of the plasmonic and photonic components were balanced at κ = 0; however, for the nonzero absorption, the photonic component was much more affected than the plasmonic one. At κ = 0.05, the energy contribution integrated for *z* > −70 nm (above the top surface of the nanopillars) dropped below 30%. Despite the fact that material absorption in the overlay can cause a considerable loss of energy in this mode, the quality factor $Q_{h}$ of the hybrid modes was less affected by the absorption than the quality factor of the reference photonic mode $Q_{p}$ (Figure 4b). Another important factor, the mode volume *V*, also remained quite stable against the material absorption (Figure 4c). The robustness of *Q* and *V* in the nonzero absorption case resulted from the plasmonic component, where the energy loss was already accounted for by the high κ number of silver. The *Q* and *V* may have crucial meaning when using the designed metasurface for active device operation, as it governs the strength of the Purcell enhancement of spontaneous emission in photonic and plasmonic structures.

## 4. Discussion

There are two major advantages of the photodetection process that result from the photonic and plasmonic natures of the designed metal/perovskite heterostructure. On the one hand, the intensity enhancement at the junction interface that results from SLR will increase the absorption in the depletion zone, which is crucial for generating the photocurrent. Also, this effect will be scaled up by the increased surface area of the junction as compared with a planar junction geometry. The effect of the increased area on the junction performance was already studied in the past [29,30]. On the other hand, the photonic component, in the form of a guided Bloch mode, might be of no less importance. Although its direct contribution to the number of photogenerated carriers will not be significant, it will share a portion of its energy with the SLR. Considering that the device can work in an active operation scheme, where the incident light is absorbed in the overlay and re-emitted with a lower frequency, utilizing the hybrid modes can create the opportunity to scale up the thickness of the device without sacrificing the plasmonic enhancement in a thin depletion zone. In the follow-up to this work, there will be a more specific analysis of the energy transfer rate between the photonic and plasmonic components and thorough analysis of the device performance in passive and active schemes.

Regarding passive and active operation schemes, there are important differences in the design and operation principles of the device. In both cases, the absorption of infrared light at the junction interface is responsible for generating charge carriers. However, while the passive device deals with infrared radiation coming from the free space, the active device utilizes the luminescence generated in the overlay. In the first case, the coupling of incident light to guided modes of the metasurface can only be achieved through interaction with the nanopillar array to match the $k_{\left|\right|}$ of the incident light and guided mode. This might require tailoring the design of the nanopillar array to provide a sufficient coupling efficiency and bandwidth. In the latter case, the internal emission of the overlay material will couple with the guided modes more easily. Also, for a passive scheme, the overlay should be designed to absorb infrared light; however, the value of κ should be kept moderate ($10^{-2}$–$10^{-1}$) in order to maintain the photonic component. Then, the chosen perovskite could be one with an electronic bandgap slightly above 1.24 eV (as results from *f* = 300 THz). Good candidates to fulfill this criterion could be the well-recognized solar cell material methylammonium lead iodide (MAPbI_3_) [31] and some of the recently developed tin-iodide- or germanium-iodide-based perovskites [32,33]. In contrast, the overlay material used for the active operation scheme should be an efficient infrared emitter with possibly small self-absorption, which is related to the Stokes shift of the material (spectral shift between the absorption and emission bands). Highly Stokes-shifted perovskites with infrared emission can be made through lanthanide doping [34] and they can be used for the indirect detection of shorter-wavelength visible light or even X-ray radiation by utilizing the radioluminescence phenomena [35]. Finally, given the very broad choice of perovskite compounds and possibility of using convenient solution-based deposition methods, such as spin coating, there is big potential in designing layered structures with multiple absorption bands, which can be used for broadband light detection. This can also involve the combination of passive and active layers for optimizing the absorption at the interface while maintaining efficient emission in the overlay. It should be noted that perovskite vulnerability to moisture or temperature is a significant obstacle in the development of perovskite-based optoelectronics, as it can limit the durability of the devices. However, continuous efforts are being made to recognize the degradation mechanisms in perovskites and develop efficient strategies to increase their stability. Perovskite thin-film devices are often encapsulated in polymers to isolate them from harmful ambient conditions [36]. Such an encapsulating layer could also be included in the photodetector design reported here without significant change to the mode characteristics. Isolating the device from the ambient atmosphere would also help to avoid the oxidation of the Ag film. Moreover, there are many recent works that have reported improvement in perovskite photodetector stability due to passivated surface defects [37] or the improved crystallinity [38,39] of the perovskite film.

## 5. Methods

All simulations were conducted using the 3D FDTD solver in Ansys Lumerical. Randomly distributed, in-plane-oriented dipole sources (dipole cloud) were used for the optical excitation of the structure. Bloch boundary conditions in the *x* and *y* directions and perfectly matched layers (PMLs) in the *z* direction were used for modeling the mode profiles in infinite structures, and the electric field was recorded by one vertical (x,z) and one horizontal (x,y) frequency domain field monitors. The band dispersion diagram was achieved by sweeping the wavevector *k* in the simulation across the Brillouin zone. The simulation time was set to 1000 fs for each value of *k*. A non-uniform mesh size was applied with a refinement to 2 nm in the pillar region. A number of randomly distributed time monitors were used for monitoring the decay of the electric field following the excitation. The Q-factors were obtained from fitting the decay curve using the Lumerical built-in functionality. The mode volumes *V* were computed as the volume where the $E^{2}$ was greater than $\frac{{E^{2}}_{max}}{2}$. In the case of finite structures, the Bloch boundary conditions in the *x* and *y* directions were replaced with PMLs that were positioned at a 3 μm distance from the geometrical borders of the structure. The two frequency domain field monitors in the *x* and *y* directions were located near the surface of the Ag patch and the top surface of the Ag nanopillars.

## 6. Conclusions

In this work, we explored the behavior of a hybrid plasmonic–photonic heterostructure and we introduced a new concept of utilizing the hybrid modes to boost the performance of photodetectors based on the metal/semiconductor junction with a Schottky barrier. The hybrid modes arise from the coupling of the guided Bloch modes propagating in the semiconducting overlay with the SLR modes supported by silver nanopillars. We optimized the design of the junction to achieve an enhancement in the optical intensity near the frequency of 300 THz. The simulation of 4 × 4 μm pixels with the designed nanostructure showed an intensity enhancement up to 5 times as compared with a planar junction design. We also investigated the effect of material absorption on the mode characteristics and we discuss the potential of using perovskite materials for both passive and active types of overlays. The demonstrated results imply that the hybrid mode operation of the device can be advantageous for overcoming the thickness limitations in plasmonic Schottky junctions. Moreover, the possible applications of our design are not limited to photodetectors but can also be used in other devices, such as nuclear batteries, which utilize Schottky junctions and can benefit from photonic and plasmonic enhancements [40].

## Data Availability

The data concerning all the results in this work are not publicly available at this moment but may be obtained from the authors upon request.
